# Overview of Poultry *Eimeria* Life Cycle and Host-Parasite Interactions

**DOI:** 10.3389/fvets.2020.00384

**Published:** 2020-07-03

**Authors:** Sara López-Osorio, Jenny J. Chaparro-Gutiérrez, Luis M. Gómez-Osorio

**Affiliations:** ^1^CIBAV Research Group, Facultad de Ciencias Agrarias, Universidad de Antioquia, Medellín, Colombia; ^2^Alura Animal Health and Nutrition, Medellin, Colombia

**Keywords:** host-parasite interaction, cell invasion, chicken coccidiosis, *Eimeria*, Intestinal lesions, oocysts

## Abstract

Apicomplexan parasites of the genus *Eimeria* are organisms which invade the intestinal tract, causing coccidiosis, an enteric disease of major economic importance worldwide. The disease causes high morbidity ranging from an acute, bloody enteritis with high mortality, to subclinical disease. However, the presence of intestinal lesions depends on the *Eimeria* species. The most important poultry *Eimeria* species are: *E. tenella, E. necatrix, E. acervulina, E. maxima, E. brunetti, E. mitis*, and *E. praecox*. Key points to better understanding the behavior of this species are the host-parasite interactions and its life cycle. The present paper reviews the literature available regarding the life cycle and the initial host-parasite interaction. More studies are needed to better understand these interactions in poultry *Eimerias*, taking into account that almost all the information available was generated from other apicomplexan parasites that generate human disease.

## Introduction

Coccidiosis is the term used to describe an enteric disease caused by infection with one or more species of *Eimeria* (1), and has a high economic impact on the poultry industry worldwide (2, 3). The etiology of this intestinal disease are pathogenic *Eimeria* species that belong to the phylum Apicomplexa, in particular *Eimeria maxima, E. tenella*, and *E. acervulina* (1). Currently, seven species of *Eimeria* are known to infect chickens and differ in pathogenicity. However, other species have been described (4). Differences between these *Eimeria* species include the invasion of specific sites of the intestine, pathogenicity and type of lesion produced (Table 1) (6).

**Table 1:** Lesions and pathogenicity of *Eimeria* spp. in chickens.

**Host**	***Eimeria***	**Location—Lesions**	Pathogenicity^*^
Chickens	*E. acervulina*	Duodenum, Jejunum. Lesions include numerous whitish, oval, or elongated patches in the upper half of the small intestine, which may be easily distinguished on gross examination.	++
	*E. brunetti*	Ileum, Rectum. The mucosa is pale and disrupted but lacking in discrete foci, and may be thickened. In severe infections, coagulative necrosis and sloughing of the mucosa occurs throughout most of the small intestine.	+++
	*E. maxima*	Duodenum, Jejunum, Ileum. It causes dilatation and thickening of the wall; petechial hemorrhage; and a reddish, orange, or pink viscous mucous exudate and fluid.	++
	*E. mitis*	Duodenum, Jejunum. Lesions are indistinct but may resemble moderate infections of *E brunetti*.	+
	*E. necatrix*	Jejunum, Caeca. Major lesions in the anterior and middle portions of the small intestine. Small white spots, usually intermingled with rounded, bright-, or dull-red spots of various sizes, can be seen on the serosal surface.	+++
	*E. praecox*	Duodenum, Jejunum. Decrease rate of growth	+
	*E. tenella*	Caeca	+++

* *, non-pathogenic; +, low pathogenic; ++, moderately pathogenic; +++, highly pathogenic*. *(5)Richard W. Gerhold, MSD Manual, https://www.msdvetmanual.com/poultry/coccidiosis/overview-of-coccidiosis-in-poultry*.

The clinical disease in broilers includes diarrhea (from mucoid and watery to hemorrhagic), reduction in weight gain and feed ingestion, and in severe cases, mortality (7). Most chickens are infected with coccidias at some point in their life, but only a few will develop clinical manifestations of coccidiosis. The clinical symptoms tend to occur in young animals, but occasionally affect adults (1, 8, 9). Challenge with low levels of *Eimeria* can stimulate the protective host immune response and this is the basis of vaccination strategies (1, 10). It has been stated that the disease only occurs if the animal is exposed to a high infective dose or host immunity is rather weak (1). Due to the self-limiting nature of the life cycle and enhanced resistance to reinfection, coccidiosis is rarely a problem in extensively raised systems, but it becomes important in closely confined and highly intensive production systems. The strategies for the control of the disease includes the use of vaccination and drugs for prophylaxis. Nevertheless, the continuous use of different coccidiostats have led to drug resistance by *Eimeria* (11).

*Eimeria* spp. exhibit high degrees of host and site specificity. This explains why any animal can host several *Eimeria* spp., each with a distinct location in the intestine. Each *Eimeria* spp. produces different host-parasite interactions generating varied symptomatology of coccidiosis (12). The first phase in the parasite cycle is the *Eimeria*-host cell interaction which leads to the massive destruction of intestinal cells (11). The understanding of this interaction and the environmental factors are key for the correct control of the disease (11). The objective of this review is to explore the poultry parasite *Eimeria* and to summarize the information available about its life cycle and the initial host cell-parasite interaction.

## The Genus *Eimeria*

**Figure 1:**
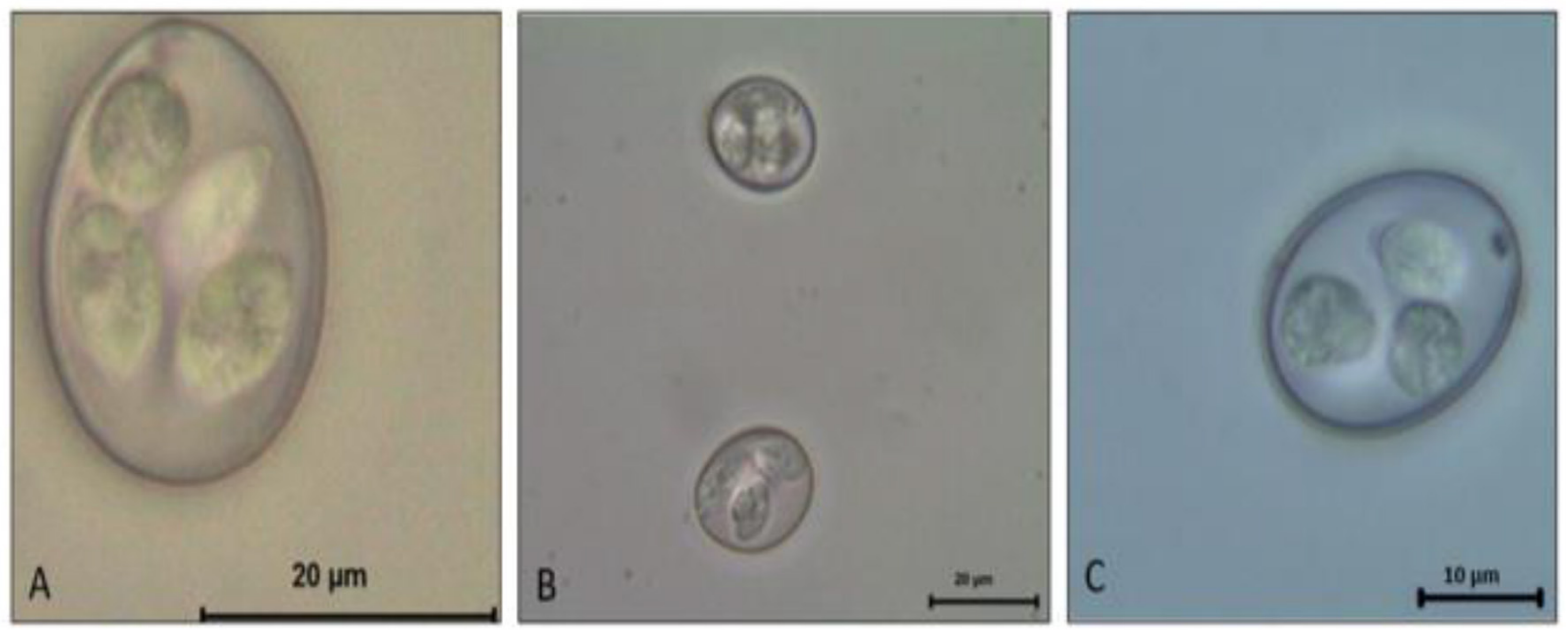
*Eimeria* spp. Sporulated oocyst. **(A)** *E. máxima*. **(B)** *E. tenella*. **(C)** *E. acervulina*.

This genus is composed of ~1,700 species, affecting both domestic mammals and birds. All *Eimeria* spp. are species-specific and therefore known as monoxenous parasites (13). The genus *Eimeria* contains the species of most economic impact for chickens. In general, all freshly shed oocysts consist of a thickened outer wall and a rounded mass with a nucleated zygote; however, once sporulation occurs the distinguishing characteristics of each species become more apparent. For the *Eimeria* genus, four sporocysts develop within the circumplasm of the oocyst, each containing two banana-shaped sporozoites (Figure 1). In contrast, the other genus *Cystoisospora* contains two sporocysts, each containing four sporozoites (12).

## Life Cycle

The *Eimeria* life cycle has basically two stages: the exogenous phase (sporogony) and the endogenous phase (schizogony and gametogony) (14). The initial infective unit of all *Eimeria* spp. is the sporozoite stage, which is a banana-shaped motile cell. The sporozoite of every apicomplexan parasite is characterized by a unique complex of structures specialized in the invasion of the host cells (15). The sporozoite is the beginning and the end of the life cycle of any coccidian (16). Sporozoites are the infective forms found in sporulated oocysts and are the result of protoplasm segmentation (16). The protoplasm (sporont) is surrounded by a resistant oocyst wall and is eliminated with the stool. Oocysts are ovoid and vary in size and shape according to the species (Figure 1).

After the exogenous phase (sporogony), sporulated oocysts can initiate replication once they are ingested orally by a susceptible host, in this case the chicken (1). Inside the intestines of the host, the sporozoites are released from oocysts under the influence of digestive enzymes and mechanical disruption. For the emergence of the sporozoite, two separate stimuli must be present: first, stress by carbon dioxide (CO_2_), which causes the rupture of the micropyle and increase in the permeability in the oocyst. This leads to a collapse of the contents of the oocyst in a hypertonic salt solution (17). The optimal concentration of CO_2_ and time of incubation differs according to the species (7). The temperature is also essential for the liberation of infective sporozoites (i.e., body temperature) (18). Secondly, the action of compounds, such as trypsin and bile (19), activate the sporozoites inside the sporocyst and digest the Stieda body generating a hole in the sporocyst membrane. Bile can either facilitate entrance of digestive enzymes through the altered micropyle into the oocyst, or can alter lipoproteins of the Stieda body of *Eimeria* oocysts (20). Although bile is not strictly necessary for activation of sporozoites, it has been demonstrated that lack of bile for many *Eimeria* spp. results in a slower release and mobility of egressed sporozoites (19, 21). Trypsin digests the sporocyst wall, along with parasite-specific enzymes secreted by activated sporozoites. Due to the continuous movement of sporozoites, the Stieda body swells and then disappears, leaving a small hole through which the sporozoites escape (21). This process is very fast and involves a strong constriction generated by the rapid movement and pressure of the sporozoite in order to go through the hole (21–25). Up to this moment of egress, no damage is done to the host. During the excystation and invasion of the host cell, the sporozoite uses its stored amylopectin for its energy requirements. Vetterling and Doran (1969) observed that during the 30 min period of excystation at 42.9°C, carbohydrate reserve levels decreased 2/3 in activated sporozoites. This is also correlated with the consumption of oxygen and lipid compounds (26, 27).

Free sporozoites infect intestinal cells of the gut and develop inside a parasitophorus vacuole (PV) into a rounded and growing organism called the trophozoite, which becomes a meront during the first merogony generation (12, 28, 29). As the sporozoite grows, the endothelial cell becomes hypertrophic and its nucleus undergoes alterations, becoming larger with an enlarged nucleolus with scattered chromatin; its cytoplasm is organized in two concentric zones and is not vacuolated appearance (30). Initially, the nucleus of the host cell has a random distribution, but then, it migrates to the periphery in order to accommodate meront development (29).

Merogony begins with multiple nucleus divisions of the *Eimeria* trophozoite without the division of the cytoplasm, resulting in the formation of ellipsoidal structures called blastophores with a peripheral layer of nuclei. The merozoite forms around each nucleus and grows radially. At the end of the phase, the division of the cytoplasm results in the formation of mononuclear spindle-shaped, motile daughter cells, known as merozoites (12). Mature merozoites I are separated by the residual body, a remnant of the blastophore (26). Once the meront is mature, the merozoites I rupture the cell and escape into the lumen of the small intestine and are most probably transported by intestinal stream to the large intestine, where merozoites I enter new cells. Merozoites I have a polar ring containing a conoid with fibers grouped in a narrow helix. Two rhoptries extend from the cone to the back of the parasite with a parallel bar in its neck. The anterior region contains abundant micronemes, 22 subpelicular tubes, of which three have granules of glycogen, many ribosomes, one or two mitochondria, a micropore and an endoplasmic reticulum (26, 31). These merozoites I enter into epithelial cells and develop into second meront stages and releasing only few merozoites II (31).

After the maturation of second meront stages, released merozoites II invade adjacent epithelial cells undergoing sexual gamogony. During the gamogony, most merozoites II develop into a single, large, mononuclear, spheroid cell, the female macrogamete. The macrogametes have characteristic eosinophilic granules [outer granule layer containing glycoproteins and an inner granule layer containing protein-rich molecules; both commonly known as “wall forming bodies” (WFB1, WFB2)]. Few merozoites II develop into large, polynucleated cells (male microgamonts) which form many spindle-shaped cells with two flagella, the microgametes (32).

The gamonts quickly generate alterations in the host cell, which distorts and loses its columnar structure (33). The pathological changes and the clinical signs associated with *Eimeria* are generated mainly by the gamonts (26), as they generate destruction of the mucous membrane of the jejunum, ileum, and caecum, causing imbalances in absorption (especially water and electrolytes) and resulting in diarrhea (12).

Thereafter, free-released microgametes fertilize surrounding macrogametes, forming the zygotes. The eosinophilic granules converge and form a resistant oocyst wall surrounding the zygote which decreases in size and becomes a sporont. The oocysts are finally released from ruptured epithelial cells and excreted with the feces into the environment (9, 12).

The un-sporulated *Eimeria* spp. oocyst excreted from the host contains a diploid sporont stage which develops further by meiosis. First, four haploid sporoblasts are generated, and enclosed by a shell thereby becoming a sporocyst. Two sporozoites are newly formed in each sporocyst. The sporont also generates a refractile polar body after meiosis (34). This exogenous sporulation process (also known as sporogony) requires optimal environmental conditions, including sufficient oxygen, moisture, and adequate temperature (16%, 23°C) (35). Sporulation seems to be a strictly aerobic process. This process requires 1–2 days. Once the sporulation ends, the metabolism and respiration of the oocyst are reduced, however, it uses its reserves of polysaccharides, and eventually becomes non-infective because the parasite runs out of energy to carry out the process of final endogenous excystation in the gut lumen (17). Sporulated oocysts may survive for long periods outside the host, depending on environmental factors. Oocysts are resistant to some disinfectants commonly used around livestock but are killed by freezing or high environmental temperatures.

## Mechanism of Invasion

To generate disease, apicomplexan parasites first need to invade susceptible host cells. To achieve this, the process of recognition and initiation of the infection are key points that might be used as targeting factors for potential treatment. Currently, there are numerous studies on this process for parasites such as *Toxoplasma gondii* and *Plasmodium* spp. (36–40). However, information on *Eimeria* spp. invasion is poor, and there are still gaps in understanding of how infection occurs (15). Sequentially, we can divide the process of invasion of target cells into 5 essential steps, allowing for a better understanding of this event.

## Recognition of the Target Cell

Invasion requires recognition and interaction of the sporozoite with the host cell. *Eimeria* spp. can only complete its life cycle and to produce oocysts shed within the feces in its specific host; however, for this specific process the parasite requires a series of stimuli (22, 41, 42). Between 1929 and 1954 a large number of studies were carried out trying to infect different host species with several types of *Eimeria*, however, most of the experiments were unsuccessful, and only experimental infections of chickens with the turkey species (*E. gallopavonis* and *E. meleagridis*) were successfully achieved (43). These observations suggested that some recognition molecules were probably necessary in order for sporozoites to enter specific host cells.

Although host cells do not have an active role in the physical process promoting entry of the parasite into the cell, they provide appropriate surface molecules and receptors, or secrete metabolites which are believed to initiate attraction or activation of apicomplexan parasites and thereafter to initiate their recognition (15). *In vivo, Eimeria* spp. show a high degree of specificity of host cells for their development. *Eimeria* spp. usually infect a limited number of host cells, and specifically a portion of an organ or system (44, 45). This specificity may be associated with unique conditions of the intestinal lumen, such as pH, enzymes, mucous, metabolites, concentration of nutrients, microbes etc. (46).

The motility, structure and secretions of sporozoites allow them to penetrate the cell, however, there is evidence that the host cell also provides characteristics which are key to host cell infection (47–49). Among these, there are some cell-surface molecules of the intestinal epithelium that act as receptors or recognition sites for the sporozoites. This last was demonstrated by *in vitro* studies in which the invasion of the parasite was inhibited with a variety of compounds that altered the host cell membrane. Some examples of these compounds are cationic molecules, enzymes and protease inhibitors (44, 50–56).

More specific evidence has shown the presence of parasite antigens that bind to molecules present on host cell surface. Antigens of 22, 31, and 37 kDa, membrane glycoconjugates, epitopes of host cell and sporozoites, were identified as receptors and ligands. However, their inhibition does not completely impair the invasion process of the parasites, so other mechanisms must be involved (15). Some studies showed that *E. adenoeides* sporozoite antigens bind to specific components of a host cell. Augustine (1989) developed a monoclonal antibody directed to a 40 kDa antigen of the sporozoite, which markedly decreased cell invasion, thus testing the hypothesis of at least one specific receptor for invasion (57).

To date, it is believed that the mechanisms of invasion are similar for all apicomplexan parasites, however ligands/receptors may change between different species. The recognition of glycosylated groups, such as heparan sulfate and chondroitin sulfate on host cells, seem to be the rule, and may be responsible for differences in target cell specificity (58–60).

Consistently, some membrane glycoproteins have also been identified as potential cellular receptors for invasion. For several protozoa it has been proposed that adhesion is mediated by binding to lectin receptors, since it is observed that the distribution of carbohydrate residues on the luminal surface of the intestine is different according to the region (61, 62). Concerning the selection of host cells and the invasion of cells *in vitro* however, there is neither host- nor cell type-specificity, since many cell types can be infected by sporozoites. This behavior in principle had been reported earlier by some authors (63, 64).

## Movement

The invasive stages of apicomplexan parasites have a complex of specialized structures (eg., conoid, polar ring, apicoplast) and organelles attached to their membranes (65). This complex is located at the anterior end of the parasite and the excreted substances from the complex are essential for the recognition, adhesion and invasion of the host cell. Previously it was believed that the internalization of parasites occurred by passive phagocytosis, however, an active participation of the parasite in the process has been demonstrated (47, 48, 66).

Although the sporozoites can move by gliding, flexing and rotating, they do not have visible organs of locomotion, such as cilia, flagella or pseudopods. The function of the apical complex is associated with penetration into the host cell and the creation of an intracellular environment suitable for the growth of the parasite (47, 67).

The apical complex is composed of unique secretory elements (micronemes and rhoptries) and structural elements (polar rings and conoid) (68). During the active process of cell invasion, the contents of the secretory organelles are released forming a mobile union that allows the formation of the parasitophorous vacuole. The conoid is surrounded by polar rings composed of microtubules and is believed to be the mechanical support of host cell invasion (69–74). In addition, the content of the rhoptries together with the dense granules reprogram cellular functions, such as the cellular immune response (75).

The sporozoites recognize, contact and enter the cell through a circular sliding movement (gliding) (76). This movement is essential for invasion both *in vivo* and *in vitro*. In the *in vivo* setting, the sporozoites excyst from the oocyst in the intestine of the host, and subsequently migrate to the intestinal lumen where they make contact with host epithelial cells where invasion occurs (77). Once this first contact is made the sporozoite penetrates the cell with its apical complex machinery. In the *in vitro* model, gravity helps sporozoites to achieve contact with host cells, since they usually grow as monolayers, nevertheless it is known that gliding motility is essential for invasion (40). Parasites can adjust their gliding motility motor to activate migration through different tissues, to force the invasion of cells, and under certain circumstances, to actively egress from an infected host cell. This movement is regulated by internal and external factors, with the calcium signaling cascade playing a central role in the process (67, 78–82). Detailed studies of the gliding motility show that both actin and myosin are involved in this process (the entire complex of proteins is known as the glideosome) (83–86). The primary components of this apparatus have been characterized using biochemical and molecular methods, together with immunohistochemistry and ultrastructural tests (87).

## Parasite-Host Cell Binding

The ultimate objective of gliding motility of the parasite is to establish temporary adhesion in order to generate enough traction to propel itself inside the host cell. This initial contact is mediated by adhesion molecules that are released from the micronemes toward the membrane (*pellicula*) of the parasite. Of these proteins, the most characterized is AMA1 and members of the anonymous proteins related to thrombospondin, which bind directly to the motor complex of the adhesion site (88–90).

## Invasion of the Host Cell

Once the sporozoite is attached to the cell, an invagination of the cell membrane occurs in front of the advancing parasite, which produces changes in the cell membrane. There is evidence that *Eimeria* spp. secrete materials that encourage the invagination of the membrane (91). Studies in *T. gondii* revealed that the invasion is an orchestrated process accompanied by a sequential release of micronemes, rhoptries and dense granules (83, 92).

Microneme proteins are rich in adhesive domains, similar to those found in mammals, although there is little homology between the proteins. Secreted microneme adhesins, such as TgMIC2, are translocated from the surface of the parasite by an actin-myosin motor during their entry into the cell (83, 92). The contents of the rhoptries are secreted during the invasion and promote the formation of the parasitophorous vacuole. For *T. gondii*, it is suggested that the contents of the rhoptries are responsible for preventing PV fusion with the lysosomes. Also, these proteins recruit the mitochondria and endoplasmic reticulum from the host cell (93–95).

## Establishment of an Intracellular Niche

*In vitro* studies showed that the PV membrane functions as a molecular sieve, being permeable to molecules between 1,300 and 1,900 Da. It also has transmembrane proteins which are derived from infected host cells. Further, PV does not bind to lysosomes and is rapidly associated with organelles and cellular components. It has been shown that microtubules and intermediate filaments of vimetin surround the PV within a few minutes after invasion, and that some organelles are attached to it. The latter is essential to prevent PV-lysosome union (93, 96, 97). For some apicomplexan parasites, 2 routes have been described to access the contents of cellular organelles: firstly, intimate association with organelles maintained by parasite-derived proteins of the PV and secondly, manipulation of the cytoskeleton to recruit vesicles to the PV. After 4 h p. i. between 20 and 50% of the PV is covered by host cell mitochondria and host cell ER (98, 99).

In addition, it has been proposed that mitochondria are bound to the PV due to rhoptry-derived proteins such as ROP2. ROP2 is anchored to the PV membrane by hydrophobic interactions and ionic interactions with the N-terminus of the protein (100). ROP2 contains two domains that target the mitochondrial matrix and ER domains exposed in the cytosol. The intimate contact between the organelles and the PV facilitates the transfer of some products to the parasites through channels located in the membrane. However, these channels are not yet characterized (101).

After the previously described interactions, the parasite begins massive modulation of the host transcriptome (102). The transcription of genes that have impact on host defense and development of the parasite are regulated by the activation of transcription factors. Within these factors is the NF-κB family, which comprises p50 (NF-κB1), p52 (NF-κB2), and subunits RelA, RelB, and c-Rel. These factors are usually associated with neutralizing the inhibitor molecule IκBs through phosphorylation of serine residues of leading to degradation by proteasomes. The active heterodimer is translocated to the nucleus, where transcription of genes involved in cell growth, apoptosis, and immune response begins (103). NF-κB is activated during host cell infection by various pathogens, and its activation benefits obligate intracellular apicomplexans. For *T. gondii* the activation of this factor in fibroblasts increased its survival. The anti-apoptotic machinery of NF-κB has also been reported in *C. parvum*. However, the parasite can also block the translocation of the factor to the nucleus, thus diminishing the inflammatory response (104–107). On the other hand, for *P. falciparum* the factor is also activated in the endothelial cells, generating an increase of ICAM-I expression, which is associated with the sequestration of red blood cells on the endothelium to escape phagocytosis of the spleen (108). In addition to NF-κB, proteins of the STAT family (STAT1-4, STAT5a, STAT5b, and STAT6) are activated in response to apicomplexans, leading to cytokine production. This family regulates the transcription of genes related to cell differentiation, growth and survival, together with immune response. The phosphorylation of STAT proteins is mediated by cytosine-activated Janus kinase, which produces nuclear dimerization and translocation. The activity of STAT1 is important for cellular defense mechanisms while STAT3/6 promotes the intracellular development of *T.gondii* (109–113).

## Conclusion and Perspectives

There is still a lack of information on the behavior of *Eimeria* spp. inside the host cell. Most of the available information is extrapolated from other apicomplexan parasites. On a very simplistic level, these interactions should be similar because they belong to the same family, nevertheless, as we know, the pathogeny and behavior of coccidias is different, so there must be substantial differences that have to be elucidated through *in vitro* studies. Additionally, transcriptomic studies will help to identify the proteins present in different phases of the cycle and help to understand better the behavior and possible targets for development of new drugs.

## Author Contributions

All authors listed have made a substantial, direct and intellectual contribution to the work, and approved it for publication.

## Conflict of Interest

LG-O was employed by the company Alura Animal Health and Nutrition. The remaining authors declare that the research was conducted in the absence of any commercial or financial relationships that could be construed as a potential conflict of interest.
